# Supplementary material for: Concerning the eXclusion in human genomics: the choice of sex chromosome representation in the human genome drastically affects the number of identified variants
Source: G3 (Bethesda). 2023 Jul 27;13(10):jkad169. doi: 10.1093/g3journal/jkad169 (PMC10542555; doi:10.1093/g3journal/jkad169)
Supplement: jkad169_Supplementary_Data [file jkad169_supplementary_data.pdf]

Supplementary Materials

Supplemental Figure 1: Major allele frequency plots for all SNPs in each of the three X chromosome regions that possess high sequence similarity to the Y chromosome.

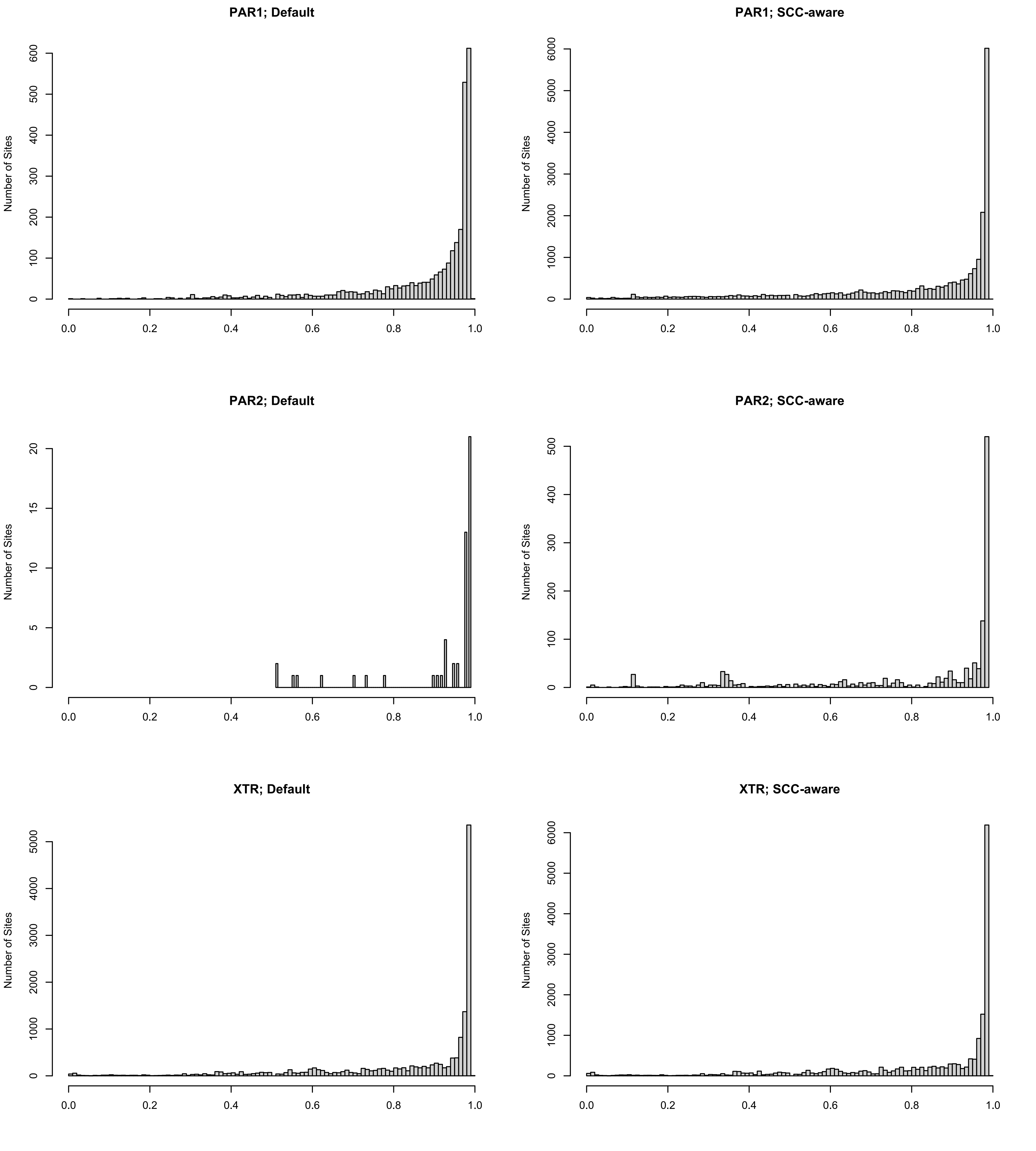
PAR1; Default
Number of Sites
0
100
200
300
400
500
600
0.0
0.2
0.4
0.6
0.8
1.0
PAR1; SCC-aware
Number of Sites
0
1000
2000
3000
4000
5000
6000
0.0
0.2
0.4
0.6
0.8
1.0
PAR2; Default
Number of Sites
0
5
10
15
20
0.0
0.2
0.4
0.6
0.8
1.0
PAR2; SCC-aware
Number of Sites
0
100
200
300
400
500
0.0
0.2
0.4
0.6
0.8
1.0
XTR; Default
Number of Sites
0
1000
2000
3000
4000
5000
0.0
0.2
0.4
0.6
0.8
1.0
XTR; SCC-aware
Number of Sites
0
1000
2000
3000
4000
5000
6000
0.0
0.2
0.4
0.6
0.8
1.0

Supplemental Figure 2: Major allele frequency plots for non-overlapping SNPs between the two reference configurations in each of the three X chromosome regions that possess high sequence similarity to the Y chromosome.

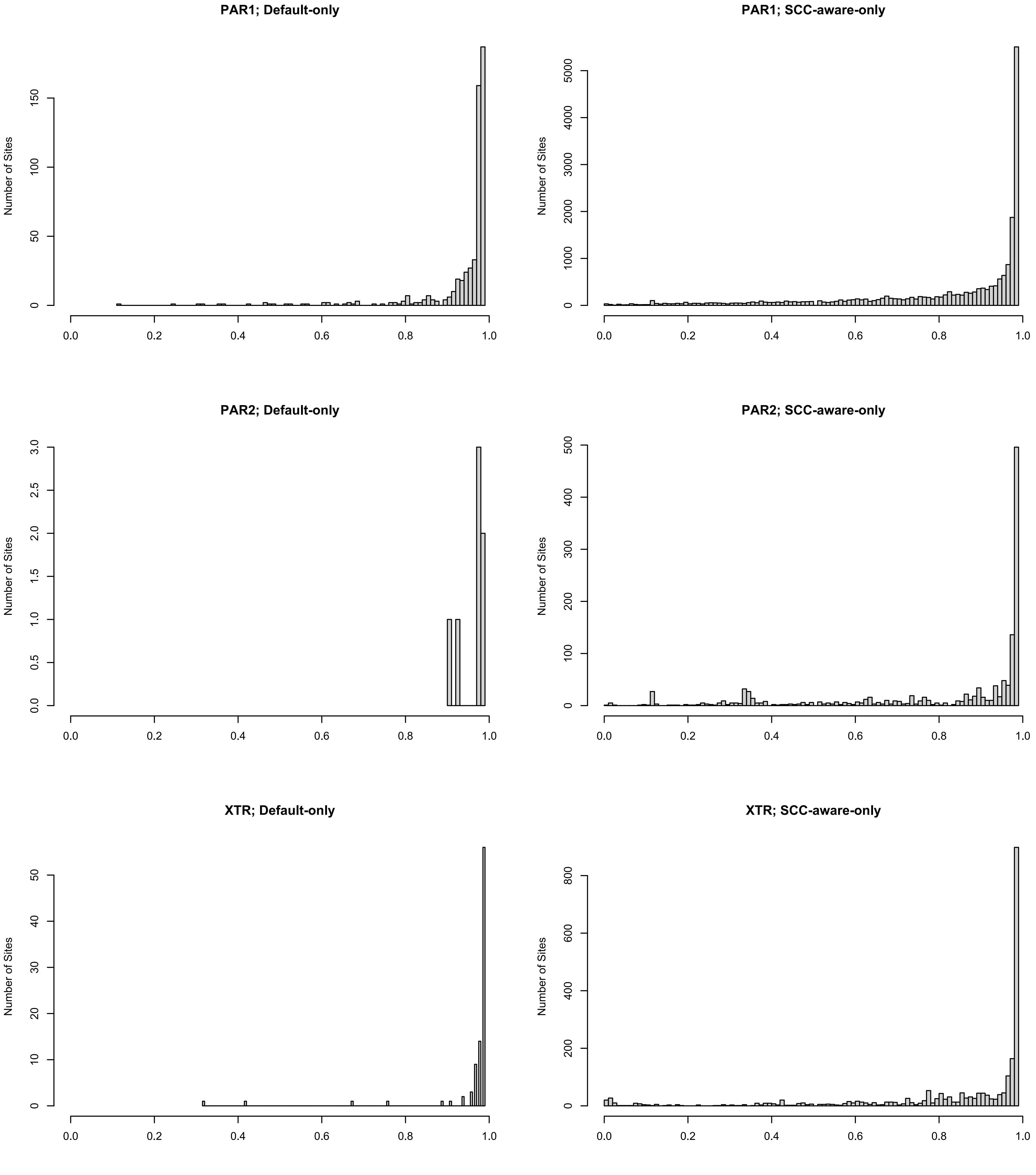

PAR1; Default-only
PAR1; SCC-aware-only
PAR2; Default-only
PAR2; SCC-aware-only
XTR; Default-only
XTR; SCC-aware-only
Number of Sites
0
50
100
150
1000
2000
3000
4000
5000
0.0
0.5
1.0
1.5
2.0
2.5
3.0
200
300
400
500
10
20
30
40
600
800
0.2
0.4
0.6
0.8

Supplemental Figure 3: Interrogating expression differences across the X chromosome. **(A)** We find that false changes in expression values are (i) nearly absent in the XTR, (ii) greatest in the PARs, and (iii) occur sporadically throughout regions with little sequence similarity with the Y. **(B)** No relationship between total transcript length and differences in expression values. There was also no observed relationship between expression levels and observed expression differences with either the **(C)** Default or **(D)** SCC-aware reference transcriptomes.

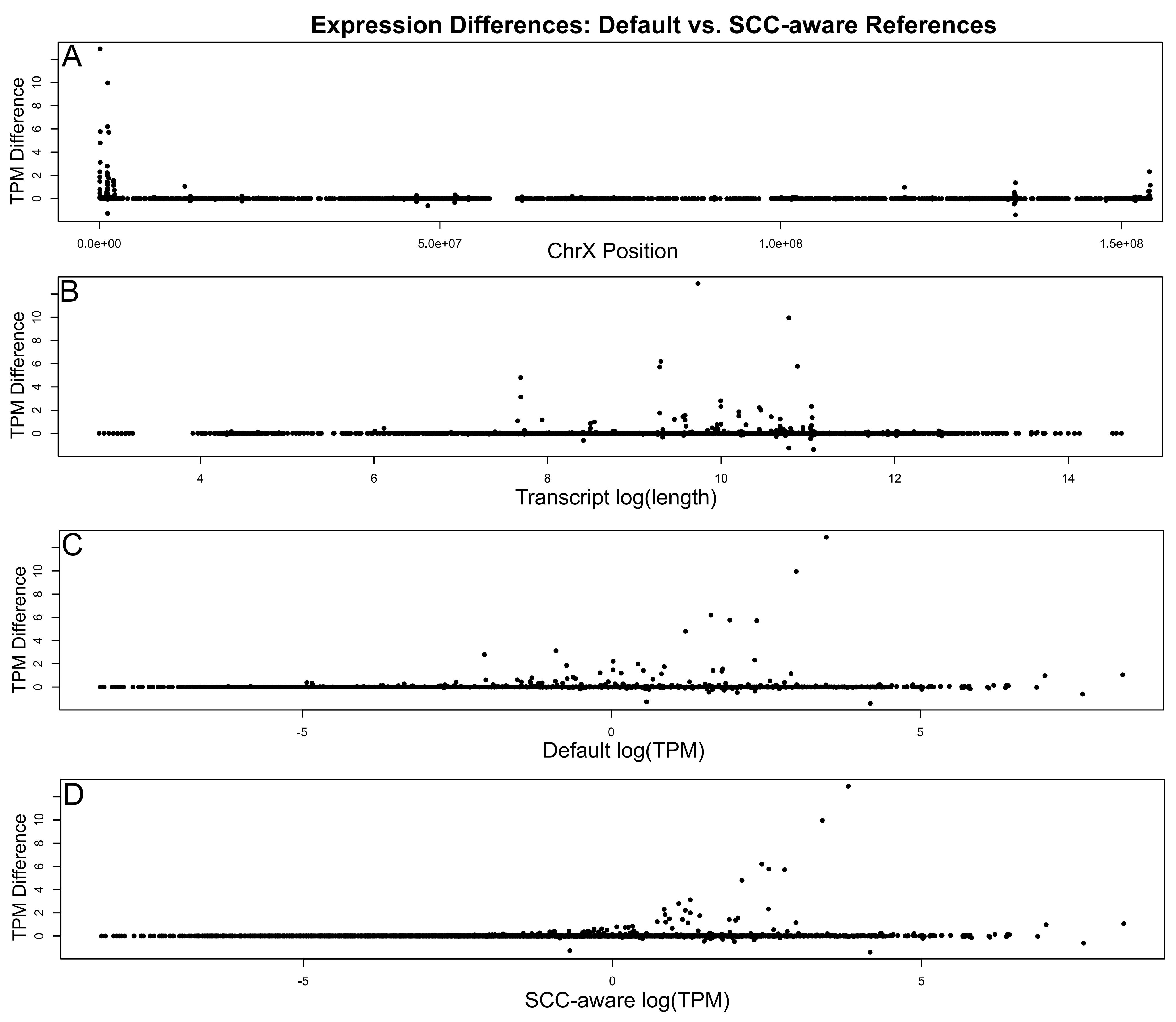
Expression Differences: Default vs. SCC-aware References
A
B
C
D
TPM Difference
ChrX Position
Transcript log(length)
Default log(TPM)
SCC-aware log(TPM)
0.0e+00
5.0e+07
1.0e+08
1.5e+08
0
2
4
6
8
10
12
14
-5
5

Supplemental Table 1: Numbers of properly paired reads aligned per-individual and their differences (Diff == SCC-aware - Default).

Sample	Default	SCC-aware	Diff
GTEX-11GSP	743,538,052	743,514,590	-23462
GTEX-11TTK	762,276,120	762,263,034	-13086
GTEX-12WSD	779,860,512	779,877,012	16500
GTEX-12ZZX	770,109,140	770,094,676	-14464
GTEX-1313W	852,501,364	852,498,722	-2642
GTEX-131XW	718,436,724	718,428,810	-7914
GTEX-13JUV	850,778,578	850,773,524	-5054
GTEX-13O3O	678,903,264	679,008,546	105282
GTEX-13OVJ	657,151,114	657,202,072	50958
GTEX-13PL6	807,524,294	807,517,724	-6570
GTEX-13QJC	752,669,274	752,663,422	-5852
GTEX-13S7M	871,498,252	871,491,502	-6750
GTEX-13SLX	713,876,242	714,000,110	123868
GTEX-13X6K	829,626,906	829,620,008	-6898
GTEX-145MI	747,642,106	747,638,062	-4044

GTEX-14BIM	807,883,826	807,878,368	-5458
GTEX-14BIN	746,761,356	746,748,214	-13142
GTEX-14LZ3	814,097,488	814,090,184	-7304
GTEX-14PJM	636,542,840	636,587,472	44632
GTEX-14PQA	793,581,622	793,576,584	-5038
GTEX-15DCD	785,001,666	784,994,964	-6702
GTEX-15DYW	769,319,014	769,312,500	-6514
GTEX-15ER7	1,032,636,384	1,032,596,560	-39824
GTEX-17JCI	722,476,160	722,468,042	-8118
GTEX-183WM	644,882,656	644,859,502	-23154
GTEX-1B933	786,036,470	786,029,174	-7296
GTEX-1CB4H	1,023,380,652	1,023,367,536	-13116
GTEX-1EWIQ	803,716,524	803,712,240	-4284
GTEX-1F48J	773,146,722	773,139,548	-7174
GTEX-1F7RK	677,735,730	677,934,982	199252
GTEX-1F88E	769,156,638	769,153,806	-2832
GTEX-1GMR8	736,925,836	736,922,506	-3330
GTEX-1GN1U	724,367,780	724,358,848	-8932

GTEX-1GZHY	777,817,298	777,808,354	-8944
GTEX-1LH75	677,035,540	677,033,288	-2252
GTEX-1OJC4	718,447,988	718,439,508	-8480
GTEX-1R46S	729,160,502	729,151,946	-8556
GTEX-N7MT	1,101,259,154	1,101,389,954	130800
GTEX-NPJ7	1,189,397,988	1,189,402,522	4534
GTEX-PWO3	1,505,297,004	1,505,284,312	-12692
GTEX-Q2AG	1,460,521,486	1,460,507,080	-14406
GTEX-QDT8	734,468,868	734,467,724	-1144
GTEX-QVJO	936,001,950	935,995,918	-6032
GTEX-QVUS	1,022,722,188	1,022,716,138	-6050
GTEX-RU72	951,784,008	951,778,084	-5924
GTEX-T2IS	894,646,420	894,641,480	-4940
GTEX-TSE9	882,487,064	882,481,826	-5238
GTEX-WWYW	1,004,805,488	1,004,799,550	-5938
GTEX-X4EP	940,866,246	940,860,976	-5270
**Average:**	**838,995,112**	**839,001,663**	**6551**

Supplemental Table 2: Numbers of aligned reads with a mapping quality score of 0 per-individual and their differences (Diff == SCC-aware - Default).

Sample	Default	SCC-aware	Diff
GTEX-11GSP	52,587,267	51,963,148	-624119
GTEX-11TTK	57,280,032	56,705,681	-574351
GTEX-12WSD	63,284,582	62,699,648	-584934
GTEX-12ZZX	63,128,131	62,555,073	-573058
GTEX-1313W	56,811,303	56,257,030	-554273
GTEX-131XW	49,082,779	48,572,485	-510294
GTEX-13JUV	58,887,315	58,332,639	-554676
GTEX-13O3O	59,712,155	59,168,081	-544074
GTEX-13OVJ	52,722,912	52,207,406	-515506
GTEX-13PL6	58,661,009	58,119,562	-541447
GTEX-13QJC	54,114,368	53,593,977	-520391
GTEX-13S7M	54,699,099	54,117,601	-581498
GTEX-13SLX	59,019,631	58,446,241	-573390
GTEX-13X6K	56,917,952	56,386,489	-531463
GTEX-145MI	52,799,420	52,298,758	-500662

GTEX-14BIM	61,610,959	61,030,765	-580194
GTEX-14BIN	43,207,035	42,559,653	-647382
GTEX-14LZ3	62,991,712	62,403,932	-587780
GTEX-14PJM	49,534,691	48,982,854	-551837
GTEX-14PQA	50,100,257	49,540,704	-559553
GTEX-15DCD	47,669,993	47,063,323	-606670
GTEX-15DYW	43,796,294	43,232,473	-563821
GTEX-15ER7	74,279,573	73,529,304	-750269
GTEX-17JCI	68,252,280	67,695,300	-556980
GTEX-183WM	50,108,997	49,655,847	-453150
GTEX-1B933	54,902,828	54,367,966	-534862
GTEX-1CB4H	75,562,943	74,817,585	-745358
GTEX-1EWIQ	55,647,825	55,080,569	-567256
GTEX-1F48J	56,413,907	55,844,076	-569831
GTEX-1F7RK	56,474,139	55,910,411	-563728
GTEX-1F88E	28,710,651	28,338,932	-371719
GTEX-1GMR8	51,148,341	50,661,769	-486572
GTEX-1GN1U	55,599,531	55,075,668	-523863

GTEX-1GZHY	58,201,555	57,655,445	-546110
GTEX-1LH75	33,992,212	33,621,491	-370721
GTEX-1OJC4	40,547,630	39,981,337	-566293
GTEX-1R46S	44,843,832	44,371,247	-472585
GTEX-N7MT	82,630,657	81,661,679	-968978
GTEX-NPJ7	89,262,358	88,282,682	-979676
GTEX-PWO3	100,949,535	99,828,089	-1121446
GTEX-Q2AG	99,396,799	98,307,124	-1089675
GTEX-QDT8	24,714,409	24,395,809	-318600
GTEX-QVJO	56,956,907	56,279,207	-677700
GTEX-QVUS	67,919,175	67,198,696	-720479
GTEX-RU72	67,284,764	66,662,729	-622035
GTEX-T2IS	52,908,632	52,188,901	-719731
GTEX-TSE9	58,283,906	57,678,080	-605826
GTEX-WWYW	65,204,859	64,483,930	-720929
GTEX-X4EP	57,656,597	56,997,932	-658665
**Average:**	**57,887,219**	**57,281,823**	**-605396**

Supplemental Table 3: Per individual number of SNPs called noting no outlier individuals suggesting potentially false positives. Diff=SCC[-aware]-Def[ault]

Sample	chr8 (Def)	chr8 (SCC)	chr8 (Diff)	chrX (Def)	chrX (SCC)	chrX (Diff)
GTEX-11GSP	170326	170324	-2	93320	99972	6652
GTEX-11TTK	175931	175921	-10	88627	95224	6597
GTEX-12WSD	226710	226708	-2	148019	156376	8357
GTEX-12ZZX	178513	178513	0	94381	100696	6315
GTEX-1313W	174948	174945	-3	93046	99266	6220
GTEX-131XW	177765	177766	1	92975	99458	6483
GTEX-13JUV	237761	237761	0	156218	164340	8122
GTEX-13O3O	177356	177337	-19	97231	103865	6634
GTEX-13OVJ	176250	176242	-8	92377	98675	6298
GTEX-13PL6	184386	184384	-2	99694	106357	6663
GTEX-13QJC	174202	174198	-4	94956	101369	6413
GTEX-13S7M	176781	176773	-8	97856	104586	6730
GTEX-13SLX	227689	227679	-10	158971	167289	8318
GTEX-13X6K	176829	176833	4	98562	105018	6456

GTEX-145MI	180230	180221	-9	96266	102756	6490
GTEX-14BIM	174321	174318	-3	94983	101611	6628
GTEX-14BIN	175646	175637	-9	92269	98852	6583
GTEX-14LZ3	168114	168112	-2	97801	104341	6540
GTEX-14PJM	179596	179590	-6	92756	99146	6390
GTEX-14PQA	174755	174754	-1	96647	103237	6590
GTEX-15DCD	176549	176548	-1	97316	103664	6348
GTEX-15DYW	174323	174313	-10	92837	99687	6850
GTEX-15ER7	176037	176024	-13	95542	101525	5983
GTEX-17JCI	172198	172194	-4	89354	95742	6388
GTEX-183WM	173002	173002	0	99420	105737	6317
GTEX-1B933	170274	170270	-4	98125	104067	5942
GTEX-1CB4H	177861	177864	3	89546	95366	5820
GTEX-1EWIQ	175684	175685	1	97071	103582	6511
GTEX-1F48J	175759	175758	-1	95263	101619	6356
GTEX-1F7RK	174431	174430	-1	100467	107217	6750
GTEX-1F88E	176955	176947	-8	99597	105950	6353
GTEX-1GMR8	174315	174301	-14	93455	100245	6790

GTEX-1GN1U	180183	180174	-9	101162	107631	6469
GTEX-1GZHY	176848	176844	-4	94084	100236	6152
GTEX-1LH75	177424	177426	2	96356	102311	5955
GTEX-1OJC4	173138	173136	-2	94733	101247	6514
GTEX-1R46S	175986	175985	-1	91872	98609	6737
GTEX-N7MT	173229	173226	-3	94344	101030	6686
GTEX-NPJ7	174748	174756	8	96675	102994	6319
GTEX-PWO3	168746	168746	0	92403	98820	6417
GTEX-Q2AG	176197	176193	-4	95794	102046	6252
GTEX-QDT8	178424	178426	2	98435	104887	6452
GTEX-QVJO	167814	167813	-1	89697	95740	6043
GTEX-QVUS	177249	177249	0	93340	99673	6333
GTEX-RU72	179638	179638	0	98649	105135	6486
GTEX-T2IS	177434	177434	0	96054	102236	6182
GTEX-TSE9	172480	172478	-2	97895	104231	6336
GTEX-WWYW	178286	178293	7	94301	100727	6426
GTEX-X4EP	172029	172029	0	94240	100860	6620
**Average:**	**178884.7**	**178881.6**	**-3.1**	**98877.2**	**105413.2**	**6536.0**

Supplemental Table 4: List of ASE transcript dropouts not recovered using the Default genome configuration but recovered using the SCC-aware reference alignment.

Chr	Start	Stop	Transcript	Source	Type	Gene
X	104530	131585	rna-NM_001370370.1	BestRefSeq	mRNA	PLCXD1
X	109615	131585	rna-NM_018390.4	BestRefSeq	mRNA	PLCXD1
X	1116125	1155344	rna-NM_001161531.2	BestRefSeq	mRNA	CSF2RA
X	1116125	1159654	rna-NM_001161529.2	BestRefSeq	mRNA	CSF2RA
X	1116125	1172713	rna-NM_001379161.1	BestRefSeq	mRNA	CSF2RA
X	1116125	1174802	rna-NM_001379159.1	BestRefSeq	mRNA	CSF2RA
X	111712	131585	rna-NM_001370373.1	BestRefSeq	mRNA	PLCXD1
X	1229797	1235760	rna-NM_001636.4	BestRefSeq	mRNA	SLC25A6
X	1393021	1403889	rna-NM_005088.3	BestRefSeq	mRNA	AKAP17A
X	1396844	1441907	rna-NM_004043.3	BestRefSeq	mRNA	ASMT
X	1.54E+08	1.54E+08	rna-NM_001394353.1	BestRefSeq	mRNA	SPRY3
X	1.54E+08	1.54E+08	rna-NM_005840.4	BestRefSeq	mRNA	SPRY3
X	1.54E+08	1.54E+08	rna-NM_001145149.3	BestRefSeq	mRNA	VAMP7
X	1.54E+08	1.54E+08	rna-XM_047447120.1	Gnomon	mRNA	LOC124908551

X	2304267	2349424	rna-NM_001321367.2	BestRefSeq	mRNA	CD99
X	2304267	2354297	rna-NM_001122898.3	BestRefSeq	mRNA	CD99
